# The embryo culture media in the era of epigenetics: is it time to go back to nature?

**DOI:** 10.1590/1984-3143-AR2021-0132

**Published:** 2022-04-20

**Authors:** Pilar Coy, Raquel Romar, Jon Romero-Aguirregomezcorta

**Affiliations:** 1 Physiology Department, Universidad de Murcia, International Excellence Campus for Higher Education and Research (Campus Mare Nostrum), Murcia, Spain; 2 Institute for Biomedical Research of Murcia, Murcia, Spain

**Keywords:** uterine fluid, oviductal fluid, assisted reproduction, beckwith-wiedemann syndrome, large offspring syndrome

## Abstract

This review is intended to draw attention to the importance of the culture media composition on the health of the embryos, fetuses, newborns, and adults derived from assisted reproductive technologies (ART). Although current research and industry trends are to use chemically defined media because of their suitability for manufacturing, commercialization, and regulatory purposes, compelling evidence indicates that those media fail to adequately account for the biological demands of early embryogenesis. Here, we list the main undesirable consequences of the ART described in the literature and results we and others have obtained over the past decade exploring an alternative and more natural way to support embryo growth *in vitro*: inclusion of endogenous reproductive fluids as additives in the ART culture media for pigs, cows, and humans. This review systematically assesses the pros and cons of using reproductive fluid additives, as well as the requirements to implement this approach in the future.

## Introduction

Assisted reproduction represents one of the­ most impactful technological revolutions of the past 50 years and today affects millions of people and animals worldwide each year (Viana, 2020; World Health Organization (WHO, 2021). While initial *in vitro* fertilization (IVF) procedures required the use of biological supports such as plasma (Lewis and Gregory, 1929), blood clots (Kuhl, 1941), or serum (Chang, 1959) to promote gametes interaction and zygote development, current commercial media tend to remove any protein or organic components from their composition. The battle between use of chemically defined media and those containing undefined organic fluids seems to have been won by proponents of the former. But, at what cost? It seems clear that healthy offspring can be produced using chemically defined media and forecasts estimate that the world will be inhabited by more than 157 million people derived from at least one assisted reproduction technology (ART) by 2100 (Faddy et al., 2018). However, compelling evidence also supports the hypothesis that the current composition of media to which gametes and embryos are exposed during the week they spend in a Petri dish before embryo transfer, has concerning consequences (Ménézo and Elder, 2020). In this review, we will delve deeply into the description of this problem, pose possible solutions to overcome this challenge, and raise evidence supporting our preferred solution. We will also discuss the divergence of this approach from commercial and research trends, and therefore, the unfortunate likelihood that this solution will not be implemented rapidly.

## The problem: are all animals and humans derived from assisted reproduction healthy?

If we were asked about the number of children born so far through assisted reproduction, the answer, as always, would depend very much on the source consulted, but we could say without being too wrong that about 8 million children have been born worldwide through an ART procedure (Roseboom and Eriksson, 2021). If the question now were how many of these ART offspring are healthy, that would be much more difficult to answer precisely. However, different studies have identified an increased risk of genetic and epigenetic defects in a small percentage of ART-derived children (Shechter-Maor et al., 2018; Hattori et al., 2019). These defects translate into diverse phenotypic traits of minor or major relevance such as a higher frequency of low birth weight (Magnus et al., 2021), obstetric complications (Storgaard et al., 2017) alterations in blood pressure or cardiovascular system in adulthood, (Guo et al., 2017), lower semen quality (Belva et al., 2016), or the appearance of certain complex syndromes, such as Beckwith-Wiedemann syndrome (BWS), Angelman syndrome (AS), Prader-Willi syndrome (PWS) or Silver Russell syndrome (SRS) (Hattori et al., 2019). Underlying these undesirable outcomes is the question of molecular mechanisms that are causative in ART-derived children. At their root, many of these symptoms and syndromes may be caused by the alteration in the DNA methylation patterns responsible for imprinting (Hattori et al. (2019) and some others). More specifically it has been observed that some imprinted genes such as *H19*, *IGF2R*, *PEG1*, *MEST, PLAGL1,* and their respective imprinting control regions (*KvDMR*) in different tissues, present an altered DNA methylation pattern, which normally leads them to lose their proper imprinting status. Specifically, the normal monoallelic and parent-of-origin-specific expression pattern (reviewed by Mangiavacchi et al., 2021) is altered. For imprinted genes, this monoallelic expression is necessary for the normal growth and development of the fetus and is considered a mechanism to balance parental resource allocation to the offspring (Ishida and Moore, 2013).

Among data obtained from animal ART, results obtained in the bovine species, the closest animal model to be compared with humans in terms of reproductive function, are incredibly similar. According to the data collected by the International Embryo Technology Society (IETS) over the past 20 years, more than 16 million bovine embryos have been transferred worldwide (Viana, 2020). Problems observed in bovine ART offspring (Siqueira et al. (2019) are most frequently related to embryonic loss, implantation failure, alterations in placental and fetal organ gene expression, a higher incidence of spontaneous abortion and neonatal mortality, difficulty calving, and also adult-onset problems similar to those described in humans in terms of general homeostasis and metabolism, resulting in cows with reduced milk, fat, and protein yield. Moreover, bovine ART offspring exhibit a higher incidence, although not precisely quantified, of the so-called Large Offspring Syndrome (LOS) or Abnormal Offspring Syndrome (AOS). Unfortunately, data in the literature on the incidence and subsequent economic losses for the farmers caused by LOS are very scarce. However, a recent report derived from extensive communications with breeding farms estimated that this syndrome affects around 10% of animals derived from assisted reproduction (Rivera et al., 2021).

As for the underlying molecular mechanisms of LOS, like in humans, it appears that global loss of imprinting leading to a monoallelic expression of key genes is causative (Chen et al., 2015). Moreover, a large number of loci are similarly impacted in both human BWS and the bovine LOS. This correlates with striking similarities in phenotypes: in both cases, it is common for affected individuals to present with increased body size, enlarged tongue, defects in the closure of the abdominal wall, visceromegaly, hypoglycemia, polyhydramnios and, in the case of humans, an increased probability of certain types of cancer from the age of 8 years (unknown in bovine species due to the lack of studies on the subject), respiratory and sucking difficulties as well as sudden prenatal death (Chen et al., 2013; Mangiavacchi et al., 2021). It should be noted, though, that it remains unknown if calves born by assisted reproduction will have problems in adulthood and, above all, when they grow old, because there are no populations with these characteristics to be studied. Indeed, beef cattle are typically slaughtered before the age of 2 years and dairy cattle before the age of 5 years, whereas their normal lifespan is 20 years. Therefore, the data available to date supports the strong conclusion that BWS/LOS and different phenotypical abnormalities in ART-derived human/bovine embryos are more frequent than in their naturally conceived counterparts and that a loss of imprinting during preimplantational development appears to be a common, causative mechanism.

## The solution: does nature hold the clues?

**Figure 1:**
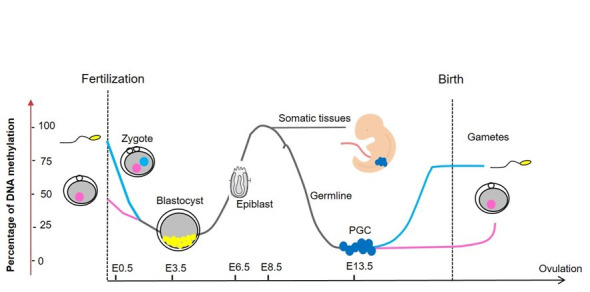
Schematic representation of the two waves of DNA methylation reprogramming in the life cycle, showing the drop in the percentage of DNA methylation from gametes to blastocyst stage. PGC: Primordial Germ Cells. E0.5, E3.5…represents days post-fertilization in the mouse.

Once the problem has been identified, possible solutions should be proposed. From our point of view, a useful approach would be to adapt the protocols used in assisted reproduction to more closely emulate nature such that the conditions to which gametes or embryos are exposed in the laboratory are as stress-free as possible. The rationale behind this option is that the sensitive period for the problematic ART outcomes is during early embryonic development and at birth. During the days-long period of *in vitro* culture, the embryonic epigenome undergoes significant reprogramming (Figure 1), and the high levels of DNA methylation of the gametes drop to a minimum at the blastocyst stage in both species (Reik et al., 2001; Ivanova et al., 2020). Therefore, any event that alters the correct reprogramming of the embryo in this first week could, according to the hypothesis of the developmental origin of health and disease (DOHAD), have consequences on the gene expression and future health of the individual (Barker, 2007). This would explain the alterations observed not only during embryonic and fetal development, as we have referred to in the bovine species, but also those mentioned in humans related to cancer, metabolism, or aging. Consequently, we hypothesize that if we can offer the cultured embryos the *in vivo* equivalent temperature, gas partial pressures (CO_2_, O_2_), nutrients, etc. t, we may perfect the conditions for *in vitro* development to reduce the undesirable outcomes of ART.

## The evidence

**Figure 2:**
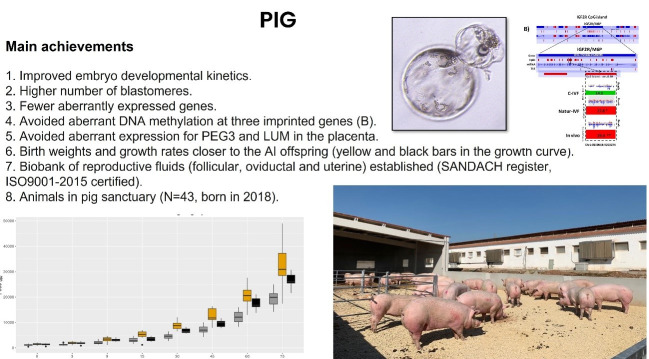
Summary of the main results derived from studies conducted in pigs to assess the effect of reproductive fluids on preimplantational embryos and the derived offspring. SANDACH: Animal by-products not intended for human consumption.

Our first glimpse of the enormous impact of *in vivo* versus *in vitro* environment for ART was the observation that the zona pellucida of oocytes collected from the oviduct resisted protease digestion for hours or days while those of oocytes matured *in vitro* were protease digestible within a few minutes (Coy et al., 2008). This difference directly impacted the success of fertilization by reducing polyspermy phenomena in the porcine species. We also determined that the composition of the oviductal fluid enzymes varied across the estrous cycle as did the effects of the fluids on the zona pellucida (Carrasco et al., 2008a, b; Coy et al., 2008). Therefore, we subsequently decided to test whether the inclusion of reproductive fluids from specific phases of the estrous cycle in the culture media would improve the epigenetic and gene expression perturbations seen in ART pig blastocysts, improve the production of live offspring and normalize gene expression patterns in the placenta. Our results (Figure 2) demonstrated improved developmental kinetics in porcine embryos, higher numbers of blastomeres in pig blastocysts, and fewer genes that were irregularly expressed compared with embryos produced with standard *in vitro* media (Canovas et al., 2017). Similarly, we found that genes which were inappropriately expressed in placentas from standard ART pig embryos, including the *PEG3* and *LUM* genes, were restored to normal expression patterns in embryos cultured with reproductive fluids (París-Oller et al., 2021a). Importantly, birth weights and growth kinetics of offspring born from fluid-cultured embryos were more similar to those conceived by artificial insemination than ART embryos cultured in the absence of these fluids (Paris-Oller et al., 2021b).

Another group of studies carried out in pigs utilized state-of-the-art technologies to measure the physiological levels of oxygen in the female reproductive tract (García-Martínez et al., 2018), as well as the temperature in the different segments of the female tract during the different phases of the estrous cycle (García-Martínez et al., 2020). These data allowed us to verify that the oxygen tension and temperature settings currently used in assisted reproduction techniques do not match normal pig physiology and when we applied these new values in the laboratory, we obtained improved results (Table 1) (García-Martínez et al., 2018, 2020).

**Table 1:** Differences between the physiological values for O_2_ and temperature recorded by García-Martinez et al. (2018, 2020) and the values routinely used during in vitro production of pig embryos.

	**Value routinely used in vitro**	**Physiological value**	**Improved parameters with the physiological values**
Temperature	38.5ºC	37ºC	Monospermy rate (20% higher)
			Blastocyst rate (15% higher)
Oxygen	20%	7%	Cleavage rate (28% higher)
			Blastocyst rate (6% higher)
			Nº cells/blastocyst (30 cells more).

Finally, we created a biobank of pig follicular, oviductal, and uterine reproductive fluids, as well as a sanctuary of animals born by assisted reproduction with or without reproductive fluids in the culture medium, to study their long-term health. Sanctuary animals will be allowed to live their normal lifespan and will be examined carefully at death.

**Figure 3:**
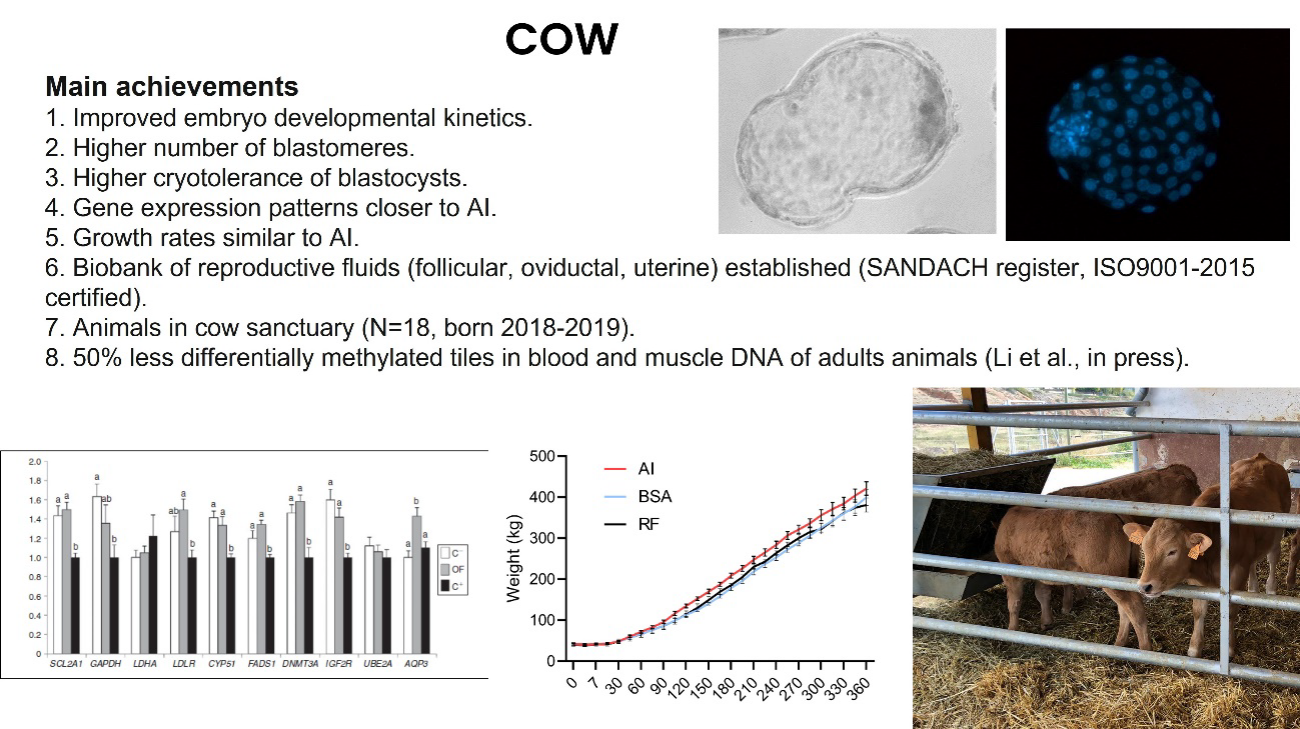
Summary of the main results derived from studies conducted in cows to assess the effect of reproductive fluids on preimplantational embryos and the derived offspring. AI: Artificial insemination; SANDACH: Animal by-products not intended for human consumption.BSA: Bovine serum albumin; RF: reproductive fluids.

In the cow (Figure 3), we also observed that embryos produced with reproductive fluids showed improved developmental kinetics, having a higher number of blastomeres, higher post-vitrification survival rates, and more similar gene expression patterns to AI embryos than embryos grown without fluids (Lopera-Vasquez et al., 2017). Indeed, the replacement of one media component, calf serum, with bovine follicular fluid had beneficial effects *on in vitro* oocyte maturation (Lopes et al., 2019). Furthermore, oviductal and uterine fluids used for embryo culture, led to living offspring whose growth rates were not significantly different from those of AI individuals (Lopes et al., 2020, 2022). By contrast, embryos grown with serum were more epigenetically similar to those collected *in vivo* than those grown with the fluids or with BSA, although a strong bias was discovered due to the embryo sex (Canovas et al., 2021). However, we have found that cows derived from embryos cultured with reproductive fluids present 50% less differentially methylated regions in blood and muscle DNA compared with artificial insemination-derived animals versus those produced with BSA instead of the fluids (Li et al., 2022). In this species, we also created a biobank of reproductive fluids and have a small number of animals in a sanctuary to study their long-term health and the effects of assisted reproductive techniques.

**Figure 4:**
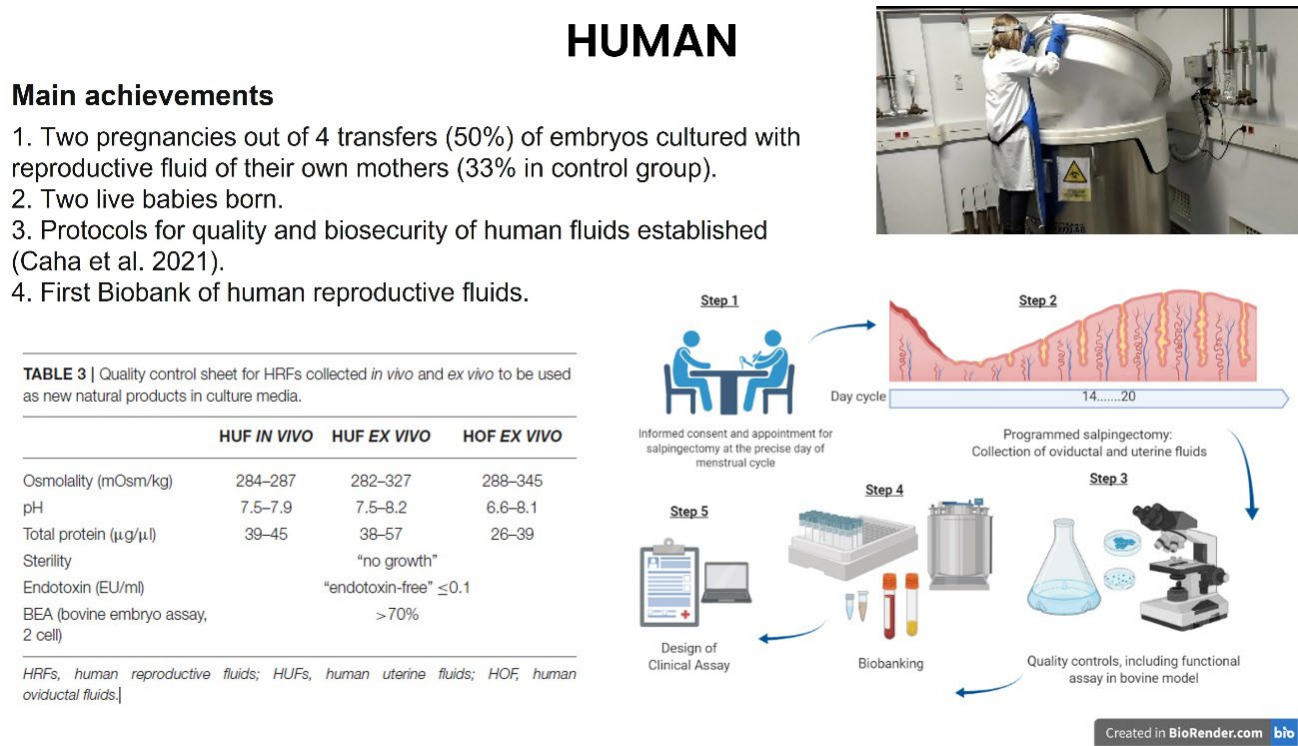
Summary of the main results derived from studies conducted in women to validate the use of reproductive fluids as safe additives for the embryo culture media.

Finally, our group completed a preliminary human clinical trial in which the embryo recipient mothers themselves donated uterine fluid to be added to the medium for culturing their own embryos (Canha-Gouveia et al., 2021). As a result, the first two children from embryos developed in culture media containing their mother's own uterine fluid were born and we also established all the necessary regulatory and biosafety conditions to create a biobank of human reproductive fluids that can be used for future research (Figure 4).

In all three species, the fluids were collected *ex vivo* (slaughterhouse specimens in pigs and cows, surgically removed organs in women) or *in vivo* (transvaginal aspiration of endometrial fluid). The fluid volumes obtained were usually between 20 and 100 microlitres, depending on the organ and the species, and the samples were immediately transported to the laboratory, where purification by centrifugation and quality control tests were performed (Canha-Gouveia et al., 2021) before storing the different batches at the corresponding biobank (human or animal). In general, fluids are added to the culture media at ~1-2% v/v and all the mentioned pieces of evidence have been obtained under these conditions. This was done because data from Lopera-Vasquez in cows (Lopera-Vasquez et al., 2017) showed that the addition of oviductal fluid at high concentrations (5%, 10%, and 25%) was detrimental to embryo development, whereas lower concentrations (1% in pigs, Canovas et al., 2017; 1.25% and 0.625% in cow, Lopera-Vasquez et al., 2017) produced higher-quality blastocysts (Figures2and3). Consequently, we undertook a series of experiments to determine the reasons for toxicity when the fluids are added at high concentrations (unpublished observations), and our results indicate that reproductive fluids are generally unstable once extracted from their physiological environment (coagulation, complement activation, proteolysis, oxidation, and formation of DNA nets). Different types of stabilizing agents are currently under study to improve the consistency of reproductive fluid batches that can be used at any concentration in the assisted reproduction protocols.

## The market trend, running far away from nature

At this point, we have identified the problem, proposed possible solutions, and the evidence of efficacy is quite compelling, but ... what does the market say, or what do experts in this field say?

Predictably, ART media use trends in industry and research are very different from what we are proposing, especially in humans. For many years, both research groups and companies manufacturing culture media have been moving towards chemically defined media, i.e. media free of proteins and additives such as fetal bovine serum. As a consequence, the current practice is to add only serum albumin, either in recombinant form in humans or obtained from serum in cattle (Sunde et al., 2016; Sena-Netto et al., 2020). But, according to a comparative study of both fluids in women doing so deprives embryos of exposure to a huge number of proteins (>1600) that would be naturally encountered in oviductal or uterine fluids, apart from the major proteins in the blood (Canha-Gouveia et al., 2019). In addition to proteins, reproductive fluids also contain other important biomolecules including microRNA and lipids, either as part of extracellular microvesicle cargo (Cañón-Beltrán et al., 2021) or dissolved in the fluids (Tribulo et al., 2019), which are also currently absent from defined culture media formulations. Indeed, an increasing number of research groups are characterizing and purifying extracellular microvesicles as promising additives to improve the efficacy of culture media (Cañón-Beltrán et al., 2021). The regulatory complexity of obtaining authorization to include extracellular microvesicles in commercial culture media, especially for human use is, however, very similar to that needed for the addition of whole reproductive fluids. Therefore, focused efforts on microvesicles could be excessive compared with simply seeking approval for use of normal reproductive fluids since the fluids contain both the extracellular microvesicles and putative beneficial molecules not included in their cargo.

Under the demographic projections made by Faddy et al. in humans (Faddy et al., 2018) and the IETS data retrieval report for bovine (Viana, 2020), the trend in the number of animals or people that will be born by assisted reproduction in the coming years is always upwards, which means that we are going to have millions of embryos that have grown during their first week of development in an environment lacking in proteins and metabolites of the natural fluids or with only very few of them. The epigenetic repercussions are clear, as we mentioned in section 5, and in humans from a well-known study from a few years ago, that showed how different culture media can affect the birth weight of children (Dumoulin et al., 2010). Further, the culture medium in which the embryo grew also continued to affect the weight of the individuals at two years of age, since, as mentioned above, low birth weights can have consequences on metabolism or blood pressure later in life (Kleijkers et al., 2014). Even more concerning is the potential for transgenerational transmission of some of these alterations, as has been already described in animal models (mice and rabbits) up to three generations (F1, F2 and F3). (Fernandez-Gonzalez et al., 2004; Garcia-Dominguez et al., 2020a, b).

As a result, there are countless publications in human ART and also in the world of cell culture in general, which indicate that serum or blood derivatives should be removed from media formulations. Despite this, the serum is still used by many scientists in specific parts of *in vitro* embryo production such as *in vitro* oocyte maturation in cattle. In fact, in a recent review on the use of bovine serum in culture media in general (not focused on assisted reproduction), it is concluded that, to date, no chemically defined substitute can replace it in most cell lines (van der Valk et al., 2018). The same conclusion was reached by Do and collaborators in a recent review in which they indicate that a suitable substitute for fetal bovine serum in culture media used for assisted reproduction in bovine species has not yet been found (Do et al., 2016).

From these observations, it can be concluded that the market trends and reality, at least in some species and protocols, follow divergent paths. And even more divergent is our hypothesis that if we want to increase the performance of assisted reproductive techniques there is nothing better than the natural environment, which we must try to emulate as much as possible. This philosophy presents a long and complicated road towards routine application because we must produce complete sets of culture media containing purified reproductive fluids from the corresponding anatomical region and from the time of the cycle corresponding to the process we are reproducing in the laboratory, be it maturation, fertilization or embryonic development. Ideally, these culture media should be ready to use with minimal manipulation to avoid inconsistencies in results and should be serum-free. Moreover, to gain the confidence of users, these media should have been tested in a bovine embryo development assay and achieve high blastocyst indices with a high number of blastomeres. They should also be free of endotoxins and any contaminating micro-organisms, and, in an ideal world, they should be available in biobanks or from commercial providers with full sanitary guarantees for use in both research and commercial embryo production. Finally, all the processes of obtaining these fluids would require specialized personnel who know and understand the reproductive physiology of each species and are capable of obtaining, processing and storing them according to the corresponding regulations.

However, we are aware that we are a long way from achieving this lofty goal. Indeed, one might question whether it even makes sense to invest time and money to achieve this goal. In other words, is the risk:benefit ratio favorable or not?

## The future: needs and risks of the “back to nature” philosophy

The obvious first step to guide decisions about use of natural reproductive fluid additives in *in vitro* embryo culture is a clear demonstration of the long-term benefits of these enriched culture media in an animal model. This requires a considerable investment of financial resources and buy-in from partner farms (for livestock species). Even with those commitments, it will take many years to see the outcomes of these studies (i.e. the birth of healthier animals or, if possible, a larger number of animals), which is a legitimate problem that must be taken into account. While we do not expect this to be the case, one possible risk is that in the end, after all of the years and money have been invested, it is possible that no tangible benefits will hold true (no benefits or the animals produced were simply equal to those obtained with conventional culture media).

A second obvious need is that, in order to demonstrate the benefit of these enriched media, they should be commercially available for many laboratories to test and use. However, researchers and commercial ART providers continue to utilize and put all their efforts into the development of chemically defined media. Yet assuming that such media do exist and are available for purchase, the new risk is that any slightest sanitary problem or poor outcome could represent a return to the past. Thus, particular care must be taken in the procurement and handling of these enriched media.

A third need is for companies to market these enriched media. This, of course, assumes there are customers for these reproductive fluid-containin media since, if there are no customers, the associated risk is that they will not make a profit and will discontinue such a product line.

Finally, a fourth need is commercial scale-up of reproductive fluid media for production of large batches that are fully tested for biological security and performance, i.e. that gave consistent results trial after trial. To meet this need, it would be necessary to source reproductive fluids (slaughterhouse tissues). However, this is a not always logistically feasible, depending on the location of slaughterhouse facilities and the numbers of animals processed per day. Alternatively, obtaining reproductive fluids from organoids derived from oviductal and uterine biopsies would be an unlimited and more consistent source for such fluids and is an interesting line of future research (Simintiras et al., 2021). Finally, the risk associated with obtaining these large batches of fluids is that the final product could be too expensive and again would be difficult to commercialize.

In conclusion, a significant amount of research will be necessary to address the main question posed in this review: How many health problems could be avoided if culture media for ART were enriched with all the components physiologically found in the female reproductive system?
